# Chemical Composition, Antioxidant, and Cytotoxic Effects of *Senna rugosa* Leaf and Root Extracts on Human Leukemia Cell Lines

**DOI:** 10.3390/ph17080974

**Published:** 2024-07-23

**Authors:** Cintia Miranda dos Santos, Debora da Silva Baldivia, David Tsuyoshi Hiramatsu de Castro, José Tarciso de Giffoni Carvalho, Alex Santos Oliveira, Paola dos Santos da Rocha, Jaqueline Ferreira Campos, Sikiru Olaitan Balogun, Caio Fernando Ramalho de Oliveira, Denise Brentan da Silva, Carlos Alexandre Carollo, Kely de Picoli Souza, Edson Lucas dos Santos

**Affiliations:** 1 Research Group on Biotechnology and Bioprospecting Applied to Metabolism (GEBBAM), Universidade Federal da Grande Dourados, Dourados 79804-970, MS, Brazil; sntos.miranda@gmail.com (C.M.d.S.); debora.s.baldivia@gmail.com (D.d.S.B.); david_hiramatsu@hotmail.com (D.T.H.d.C.); tarcisiogiffoni@hotmail.com (J.T.d.G.C.); alexsantosoliveira@gmail.com (A.S.O.); paolarocha.biologa@gmail.com (P.d.S.d.R.); jaquelinefcampos@ufgd.edu.br (J.F.C.); balogun.zhikrullah@gmail.com (S.O.B.); oliveiracfr@gmail.com (C.F.R.d.O.); kelypicoli@ufgd.edu.br (K.d.P.S.); 2 Programa de Pós-Graduação em Ciências de Saúde, Faculdade de Ciências da Saúde, Universidade Federal da Grande Dourados, Dourados 79804-970, MS, Brazil; 3 Laboratório de Produtos Naturais e Espectrometria de Massas (LaPNEM), Faculdade de Ciências Farmacêuticas, Alimentos e Nutrição (FACFAN), Universidade Federal de Mato Grosso do Sul (UFMS), Campo Grande 79070-900, MS, Brazil; denisebrentan@gmail.com (D.B.d.S.); carlos.carollo@ufms.br (C.A.C.)

**Keywords:** natural products, anticancer properties, cancer, medicinal plants, chemical profile

## Abstract

*Senna rugosa* is a species found in the Cerrado and used in folk medicine as a vermifuge and in the treatment of poisonous snakebites accidents. In this work, we identified the main secondary metabolites present in ethanolic extracts of the leaves (ELSR) and roots (ERSR) of *S. rugosa* and evaluated the potential cytoprotective effect against cellular macromolecular damage, as well as the cytotoxic properties of the extracts on the K562 and Jurkat leukemic cell lines. The identification of metabolites was carried out by liquid chromatography coupled with mass spectrometry. The antioxidant activities were investigated by direct ABTS**^•+^** and DPPH**^•^** radical scavenging methods, protection against oxidative damage in proteins, and DNA. Cytotoxic properties were investigated against healthy cells, isolated from human peripheral blood (PBMC) and leukemic cell lines. The leaf extracts contained catechin, rutin, epigallocatechin derivatives, kaempferol glycosides, luteolin, and dimeric and trimeric procyanidins, while the root extract profile showed obtusichromoneside derivatives, 2-methoxystypandrone, stilbene derivatives, naphthopyranones, and flavanone derivatives. The extracts showed antioxidant activity, with an IC_50_ of 4.86 ± 0.51 μg/mL and 8.33 ± 0.90 μg/mL in the ABTS assay for ELSR and ERSR, respectively. Furthermore, in the DPPH**^•^** assay, the IC_50_ was 19.98 ± 1.96 μg/mL for ELSR and 13.37 ± 1.05 μg/mL for ERSR. The extracts protected macromolecules against oxidative damage at concentrations of 5 μg/mL. The cytotoxicity test against leukemic strains was observed after 24 and 48 h of treatment. After 48 h, results against the K562 cell line demonstrate an IC_50_ of 242.54 ± 2.38 μg/mL and 223.00 ± 2.34 μg/mL for ELSR and ERSR, respectively. While against the Jurkat cell line, these extracts showed an IC_50_ of 171.45 ± 2.25 μg/mL and 189.30 ± 2.27 μg/mL, respectively. The results pertaining to PBMC viability demonstrated that the extracts showed selectivity for the leukemic cell lines. Together, our results reveal that the leaves and roots of *S. rugosa* have completely distinct and complex chemical compositions and expand their significant pharmacological potential in oxidative stress and leukemia conditions.

## 1. Introduction

Oxidative stress is defined as an inequality between reactive species and the body’s natural antioxidants [1,2]. Oxidative damage to macromolecules, such as proteins and nucleic acids, accelerates cellular aging and impairs DNA repair mechanisms. This increases the buildup of mutations [3,4], which is linked to the development of conditions like cancer and other diseases [5]. The deleterious effects of oxidative stress can be controlled by antioxidants, conferring protection against reactive species of endogenous or exogenous origin [6,7]. The beneficial effects of antioxidants on physiology are widely known [8]. Furthermore, antioxidants can promote pro-oxidant effects in cancer cells by activating different cell death mechanisms [9,10].

Leukemia is a hematologic neoplasm affecting the hematopoietic system’s cells [11]. Four main subtypes of leukemia are known, and diagnosis varies according to the maturity and type of cell lineage. These four main subtypes are acute lymphoblastic leukemia, acute myeloid leukemia, chronic lymphoblastic leukemia, and chronic myeloid leukemia. In addition to these main categories, there exist several less common subtypes [12]. In many cases of leukemia, translocations between chromosomes 9 and 22 are observed, leading to the fusion of the BCR and ABL1 genes. This fusion results in increased tyrosine-kinase activity [13] and the activation of several signaling pathways, such as Jak/Stat and Ras/Raf/Map-kinase, resulting in uncontrolled events during the cell cycle [14]. A lack of control over cell cycle progression can also occur due to the silencing of suppressor (p53 and PTEN) and pro-apoptotic genes (BAX) [15].

Despite advances in the treatment of leukemia, about 20% of patients have a relapse rate of the disease or adverse reactions to treatment, highlighting the importance of developing new therapies [11,16]. Consequently, a high mortality rate is recorded. In 2020, more than 19 million cases occurred worldwide, with a mortality rate of 70% [17]. In this regard, the scientific community has intensified the search for new therapeutic agents with anticancer activity of natural origin. Recent studies have demonstrated the pharmacological potential of plants from the Brazilian Cerrado, a biome with the potential for discovering new phytochemicals [18]. 

Recent pharmacological studies have shown that the crude extracts or derivatives of the *Senna* genus possess, among others, antidiabetic, antioxidant, anti-inflammatory, antitumor, and anticancer activities [19,20,21]. Several studies on medicinal plants from Cerrado have presented crude plant extracts with anticancer properties [22,23,24,25]. The extracts of these species presented a varied composition of secondary metabolites, among them gallic acid, catechin, epigallocatechin, epicatechin, kaempferol heteroside, rutin, ellagic acid, anthraquinone, piceatanol, and dimeric and trimeric proanthocyanidins. Several studies have demonstrated the effect of these compounds and their cytotoxic effects on cancer cells [26]. *Senna rugosa* (Fabaceae) is a plant species popularly known as *amendoirana*, *alcacuz bravo*, *bico-de-corvo*, *boi gordo*, and *paratudo*, where its seeds are used to treat parasitic worm infestations [27,28] and its roots are used in the treatment of poisonous snake bites [29]. Recently, Cunha et al. [30] evaluated the chemical composition of the ethanolic extract of *S. rugosa* leaves and fractions obtained from extracts and identified the presence of mainly phenolic compounds, which include flavonols, anthraquinones, and anthrones derivatives (particularly rutin, emodin, aloe-emodin, and barbaloin/isobarbaloin). In that study, antimicrobial, antifungal, and moderate antitumor activities against breast cancer cell lines were reported. There have been reports that several species of the *Senna* genus possess anticancer activities against various tumor cell lines [21,31]. 

As the species has been used by the population in the treatment of pathologies, we proposed to determine the chemical constitution of the metabolites present in the leaves and roots of *S. rugosa*. In addition, we investigated the antioxidant properties and cytotoxic activities of the ethanolic extracts of *S. rugosa* leaves (ELSR) and roots (ERSR) against chronic (K562) and acute (Jurkat) human leukemia cell lines. We used human peripheral blood cells (PBMC) in this study to determine the selectivity of the extracts on leukemic cells compared to healthy human cells.

## 2. Results

### 2.1. Chemical Composition of ELSR

**Figure 1:**
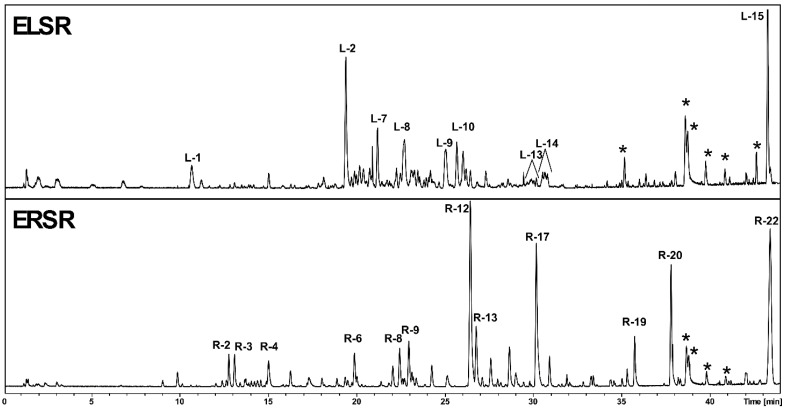
Chemical profile of ethanolic extracts of the leaves (ELSR) and roots (ERSR) of *S. rugosa* represented by chromatogram in column C-18. * Column contaminants.

The detailed phytochemical analysis of *S. rugosa*, conducted separately on its leaf extracts (Table 1—ELSR) and root extracts (Table 1—ERSR), revealed distinct chemical profiles between these two plant organs. In the leaf extracts, we identified a variety of flavan-3-ol dimers and trimers, epiafzelechin derivatives, and a range of flavonoids, including rutin, luteolin, and kaempferol 3-*O*-rutinoside, indicating a marked predominance of phenolic and flavonoid derivatives. In contrast, the root extracts presented a widely varied secondary metabolism, ranging from *C*-glycosylated chromones, notably of the obtusichromoneside class, to glycosylated naphthopyranones with skeletons compatible with rubrofusarin derivatives, stilbenoids, and possible glycosylated and sulfated flavonoids. The chromatographic profile is shown in Figure 1, while the retention times, molecular formula, and M + H are shown in Table 1.

### 2.2. Antioxidant Activity Assays

Table 2 presents the IC_50_ values for the ABTS**^•+^** and DPPH**^•^** radicals, as well as the concentrations at which the ELSR and ERSR showed maximum antioxidant activity. For the ABTS**^•+^** radical, ELSR presented an IC_50_ 1.25 times lower when compared with the reference antioxidant BHT, presenting maximum activity at the same concentrations (50 μg/mL). ERSR presented an IC_50_ 1.37 times higher than BHT, however, its maximum activity was reached at half the concentration of BHT. 

In the direct DPPH**^•^** radical scavenging assay, the IC_50_ of ELSR and ERSR were 3.5 and 5.3 times lower, respectively, than that calculated for BHT, demonstrating a higher antioxidant activity of the extracts compared to this reference compound. Both extracts showed maximum activity at concentrations 5 times lower than BHT. 

### 2.3. Protection of ELSR and ERSR against Oxidative Damage in Macromolecules

**Figure 2:**
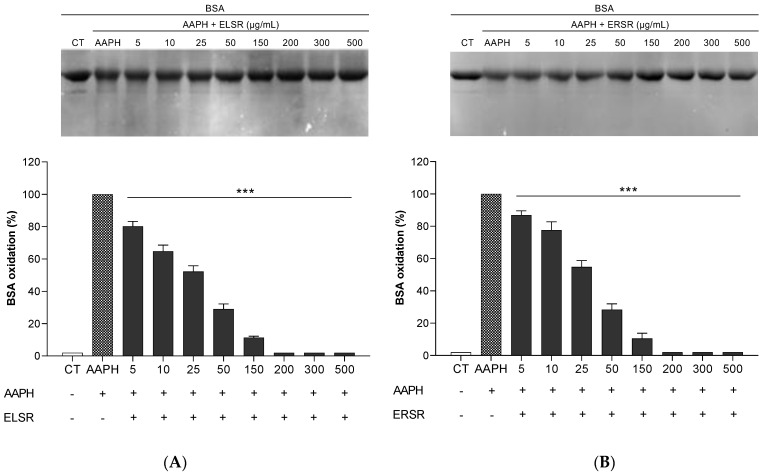
Effect of the protection promoted by the extracts against AAPH-induced oxidation of BSA. BSA was incubated with different concentrations of ELSR (**A**) and ERSR (**B**). Data present results from four independent experiments, *** *p* < 0.0001 compared to control BSA incubated with AAPH alone (AAPH). CT: BSA control.

ELSR and ERSR protected BSA protein against AAPH-induced oxidation at all of the concentrations evaluated (Figure 2). The images of the gels are representative and demonstrate the protection against oxidation after ELSR and ERSR treatments. Both extracts reduced protein oxidation resulting from AAPH exposure when compared to the AAPH control, where BSA was incubated with AAPH alone.

**Figure 3:**
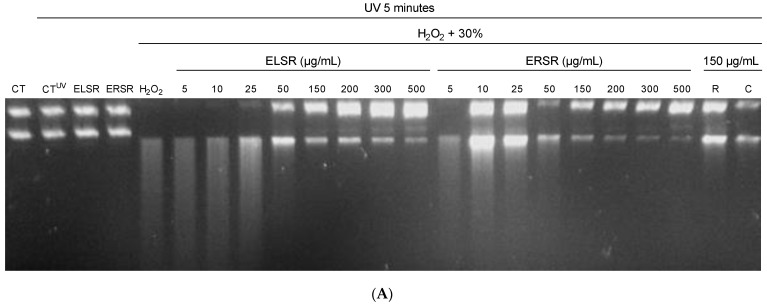

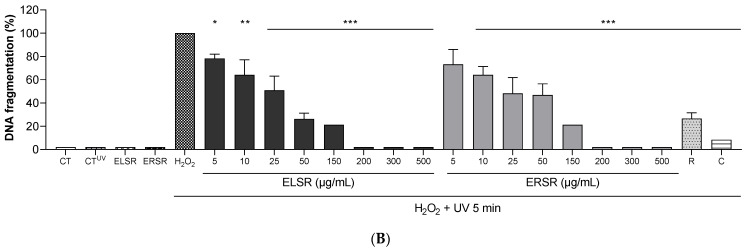
Effect of ELSR and ERSR protection against DNA fragmentation induced by H_2_O_2_ and UV radiation. Representative image of plasmid DNA bands (**A**). Percent fragmentation of plasmid DNA incubated with different concentrations (5–500 μg/mL) of ELSR and ERSR (**B**). Results expressed a percentage of DNA fragmentation. PC: Plasmid control; PCUV: Plasmid + UV control; ELSR: Plasmid + ELSR 500 μg/mL; ERSR: Plasmid + ERSR 500 μg/mL; H_2_O_2_: plasmid + H_2_O_2_ 30%; R: rutin; C: catechin. Data present results from three independent experiments,* *p* < 0.05; ** *p* < 0.01, and *** *p* < 0.001 compared to control DNA incubated with H_2_O_2_ and subjected to UV.

ELSR and ERSR were able to protect DNA from H_2_O_2_ UV-induced fragmentation in a concentration-dependent manner (Figure 3B). Figure 3A is a representative picture of the protection against fragmentation after ELSR and ERSR treatments. In the absence of extracts and at low concentrations (up to 25 μg/mL), fragmentation of plasmid DNA is observed. A reduction in fragmentation is visible from 50 μg/mL onward. The reference antioxidants rutin and catechin caused a reduction in DNA fragmentation; however, rutin was more effective in the evaluated concentration.

### 2.4. Cytotoxicity against Leukemic Strains

**Figure 4:**
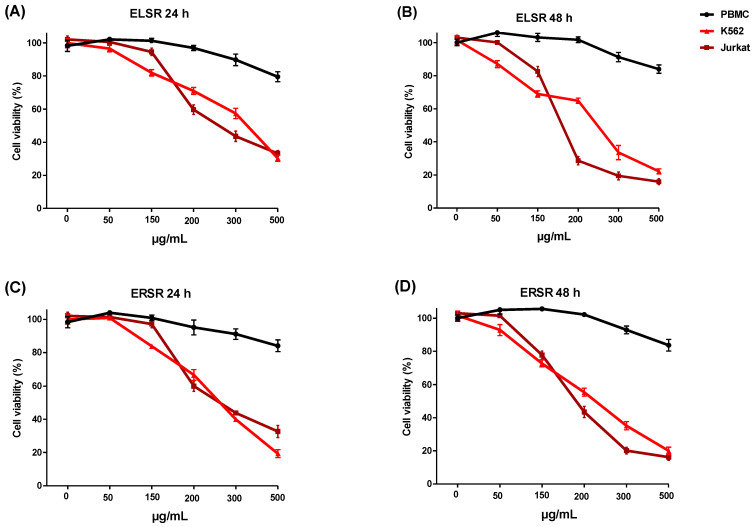
Cell viability of PBMC, K562, and Jurkat cell lines after 24 (**A**,**C**) and 48 h (**B**,**D**) of treatment with different concentrations of the ELSR and ERSR (0–500 μg/mL). Data were expressed as the means ± SEM of three independent experiments.

PBMC cells and K562 and Jurkat leukemic cell lines were treated with different concentrations of the ELSR and ERSR for 24 and 48 h. Incubation of the ELSR and ERSR (Figure 4A–D) with PBMC showed a minimal reduction in cell viability of 17% on average, but only at the highest concentration of the extracts. None of the concentrations of the extracts caused a 50% reduction in PBMC viability, suggesting that the extracts are selective for leukemic cells. The results of the solvent control (0.1% ethanol) demonstrated that the cells’ viability under these conditions was unaffected by the solvent. The results show that the extracts showed a cytotoxic effect against the leukemic cell lines in a concentration-dependent manner, with the K562 cell line being less sensitive than the Jurkat cell line, as observed through the IC_50_ values presented in Table 3.

## 3. Discussion

The phytochemical profiles of ELSR and ERSR were examined utilizing HPLC techniques to identify pertinent secondary metabolite groups and valuable chemical markers. These multi-constituent profile markers serve as tools for quality control of herbal preparations and for subsequent validity assessments.

An interesting observation in the present study is the high divergence observed in the phytochemical constituents identified between ELSR and ERSR. Furthermore, the identification for the first time of pharmacologically active metabolites from the roots of *S. rugosa*. This divergence in phytochemical profiles not only underscores the existence of a unique chemical diversity between the distinct parts of the plant but also reflects the intricate specialized metabolic networks characterizing the leaf and root tissues of *S. rugosa*.

The chemical profile of ELSR was characterized predominantly by the accumulation of flavan-3-ol derivatives, notably catechin (L-1). This compound, distinguished by its characteristic UV absorption at 280 nm and a molecular ion [M + H]^+^ at *m/z* 291.0870, was confirmed through comparison with an authentic standard. Proanthocyanidins, including derivatives such as epiafzelechin-epiafzelechin (L-4, L-5, L-6) and trihydroxyflavan-epiafzelechin derivatives (L-8, L-9, L-10, L-12), highlight the plant’s ability to synthesize complex polymeric flavonoids. These compounds were annotated by their molecular masses and distinctive MS/MS fragmentation patterns.

Flavonols, such as Rutin (L-2) and Kaempferol 3-O-rutinoside (L-7), were identified by their UV spectra, MS/MS fragmentation patterns, and comparison with an authentic standard. Flavones such as Luteolin (L-11) and its methoxylated derivative, 3-methoxyluteolin (L-13), underscore the plant’s ability to synthesize a wide variety of flavonoid structures.

The stilbenoid, 3,3′,5,5′-Tetrahydroxy-4-methoxystilbene (L-3), was detected at a low intensity and annotated by its UV absorption and molecular formula. This class has been reported multiple times within the genus [32].

Furthermore, a tetrahydroanthracene derivative (L-16), representing a less common class, was putatively characterized by its molecular weight and MS/MS fragments. It is possibly novel in the literature; due to the absence of standards, we were unable to determine its chemical structure. We recently identified a compound of this class in *S. velutina* [20].

The ERSR extract from the roots displayed a metabolite accumulation distinctly different from that observed in the leaves. Chromones prominently featured, evidenced by the annotation of obtusichromoneside derivatives (R-2, R-3, R-7) and a chromone derivative (R-7), characterized by their UV spectra and mass fragmentation patterns. These compounds have previously been reported in other species of *Senna* and *Cassia*, a closely related taxon [33].

The analysis also highlighted a significant presence of naphthoquinone and naphthopyrones, with compounds such as 2-methoxystypandrone (R-4), a putative norrubrofusarin gentiobioside (R-8) (a possible novel compound), rubrofusarin gentiobioside (R-10), cassiaside B (R-12), and rubrofusarin-O-glucopyranoside (R-14), demonstrating the root’s capability to synthesize complex naphtho-derived structures. These findings are particularly interesting due to the rare occurrence of such compounds in nature and their potential pharmacological applications.

The only compound observed in both the leaves and roots was R-6 (equivalent to L-3 in the leaf), but it demonstrated low chromatographic intensity, consistent with its observation in leaf extracts. Additionally, the detection of Flavanones and their derivatives, including hexahydroxy Flavanonol pentosyl-hexosyl derivatives (R-9, R-13), a putatively annotated hexahydroxy Flavanonol sulfate (R-11), and hexahydroxy-methoxy flavanonol sulfate (R-16), indicates a distinctive flavonoid accumulation pattern in the leaves and roots of *S. rugosa*. Some of these compounds, such as R-11 and R-15, do not match any structures described in the literature, suggesting the possibility that they are novel compounds. The structural diversity within this class highlights the intricate enzymatic processes involved in flavonoid modification.

Seven compounds of the roots remained unidentified (R-1, R-15, R-17, R-18, R-20, R-21, R-22), presenting opportunities for further research. These unknown entities, detected through their unique mass spectra and UV profiles, hint at the unexplored chemical space within *S. rugosa*, suggesting the presence of novel or rare metabolites awaiting discovery.

In summary, this detailed phytochemical investigation significantly enhances our comprehension of the chemical diversity present in *S. rugosa*, underscoring the plant’s extensive biosynthetic capacities. It sets a foundation for future research aimed at elucidating the pharmacological properties and ecological significance of these compounds, while also indicating the potential for discovering novel metabolites within this species.

Interestingly, the antioxidant and anticancer activities of most of these compounds identified in ELSR, including their molecular mechanisms, have already been proven in several studies. Notably, several in vitro, in vivo, and clinical studies have shown multiple anticancer actions of epigallocatechin, and a review of their potential therapeutic targets and their role in the therapy of various cancers has been the motive of several studies [34,35,36]. These include anti-proliferative, pro-apoptotic, anti-angiogenic, and anti-invasive activities. Another compound identified in ELSR is rutin, which exhibits a wide range of pharmacological properties. These include, among others, antiangiogenic, pro-apoptotic, antioxidant, antiproliferative, and anti-inflammatory activities. These properties may contribute to the prevention and treatment of cancer [36].

In the report by Cunha et al. [30], several peaks were observed in the hydroethanolic extract, but only one compound was identified (rutin), making comparison with our results impossible. Thus, we report the presence of different phytochemicals not previously described for this species.

In the ERSR, several pharmacologically important compounds were also identified. Of the 22 peaks, 6 could not be identified based on the current technique employed. Among the identified compounds in ERSR were the chromone derivatives, particularly the obtusichromoneside derivatives. Several chromone derivatives have been credited with significant biological and pharmacological activities, including antioxidant, antiviral, anti-inflammatory, antitumor, antimicrobial, among others [37,38,39]. Duan et al. [38] presented a comprehensive review of the antitumor activities of naturally occurring chromones. The antitumor mechanisms of these chromones include cytotoxicity (cell-cycle arrest and cell death, RNA inhibition, and inhibition of signal transducers), antimetastatic, anti-angiogenesis, and immune regulation, among others. Moreover, some have been shown to possess cancer chemoprevention potential. The other equally important secondary metabolites found in ERSR are naphthoquinones. These groups of naturally occurring bioactive compounds have been the subject of intense research on anticancer and antioxidant activities [40,41].

Several studies have demonstrated the effect of compounds present in ELSR and ERSR extracts on increasing caspase 3 activity in cancer cells, a key enzyme in the induction of apoptotic processes. The presence of rutin and luteonin in ELSR indicates the activation of caspase 3, as described by Pandey et al. (2021) [42] and Raina et al. (2021) [43] in HeLa cells, respectively. In ERSR, caspase 3 activation is probably linked to the presence of chromone derivatives (Chu et al., 2021) [44]. Specifically, chromone derivatives have been shown to induce apoptosis through the Bcl-2, Bax, and caspase 3 signaling cascades.

Furthermore, the activation of caspase 3 can occur through the activation of caspase 8 (the extrinsic pathway) and through the activation of caspase 9 (the intrinsic pathway), as observed by Pandey et al. (2021) for rutin [42]. It is therefore plausible to expect that the compounds present in the extracts can activate a single pathway for the induction of apoptosis, such as the activation of caspase 9 by luteolin or the activation of caspase 8 by epiafzelechin and procyanidins present in ELSR (Raina et al., 2021 [43]; Kaur et al., 2020 [45]; Minker et al., 2015 [46]). Thus, it is consistent to suggest that the mechanism by which ELSR and ERSR extracts exerted the final cell death effect observed in our study might be related, at least in part, to these mechanisms. The exploration of the mechanisms involved in these activities, together with in vivo antitumor activities, will be our next focus for future research.

The radical scavenging, antioxidant, and anticancer activities of these groups of compounds are widely known. These compounds together may act in synergy to account for the antioxidants and prevent oxidative damage to the macromolecules employed in this study.

The genomic mutation of DNA is an essential component in the process of cancer development. Oxidative damage and the consequent modification of DNA bases can lead to point mutations, deletions, insertions, or chromosomal translocations that, in turn, can produce oncogene activation or tumor suppressor gene inactivation [47,48]. In this context, we evaluated the potential for reactive species sequestration, antioxidant activity, DNA, and protein protective activity against oxidative damage induced by oxidizing agents.

DNA mutation is a critical step in carcinogenesis, and elevated levels of oxidative DNA damage (8-hydroxyguanosine) have been noted in various tumors, which strongly implicates such damage in cancer etiology [49]. Furthermore, the increased production of reactive oxygen species (ROS) in various cancers has been shown to play several roles, such as in the activation of pro-tumourigenic signaling, an increase in cell survival and proliferation, and driving DNA damage and genetic instability [50,51,52].

To evaluate the potential protective effect of ELSR and ERSR in mitigating oxidative damage to macromolecules, models of oxidative damage induced by hydroxide peroxide were used, and ELSR and ERSR exhibited antioxidant activity, acting in the direct capture of reactive species. Furthermore, we demonstrated that the extracts were able to promote the protection of macromolecules against oxidative damage. These results indicate that the extracts can stabilize reactive species through electron or H^+^ donation, preventing nonspecific oxidoreduction reactions that propagate in the intracellular environment [6]. The antioxidant activity of ELSR and ERSR may be related to the presence of biologically active metabolites identified in both extracts. These metabolites are widely recognized for stabilizing reactive oxygen species through hydrogen donation and binding with metal ions [53,54,55].

In addition to the effect of direct radical scavenging, ELSR and ERSR were able to protect proteins and DNA from oxidative damage. The oxidation of macromolecules by reactive species is an important deleterious effect observed at the cellular level. Protein oxidation promotes the loss of function of receptors, enzymes, and other intracellular proteins, the formation of protein aggregates, and proteolysis.

The protective effect of *S. rugosa* extracts against the oxidation of macromolecules suggests that intracellular oxidative damage can be reduced by ingesting the extracts and could present beneficial effects for human health. The intake of natural antioxidants is recognized for promoting a double beneficial effect on the organism: the protection of healthy cells against the deleterious action of reactive species and their anticancer properties. In addition to conferring protection to macromolecules, flavonoids, and naphthoquinones, among others, they can regulate the expression of oncogenes and tumor suppressor genes, control cell proliferation, metastasis, and angiogenesis, induce cell cycle arrest, and promote apoptosis in cancer cells [36,40,41,55,56,57].

Thus, based on the results obtained, ELSR and ERSR may modulate the cellular environment, promoting apoptosis activation in K562 and Jurkat cells. From this perspective, further studies are necessary to gain a better understanding of the mechanisms through which the extracts promote the death of leukemic cells.

The search for cytotoxic agents that exhibit selectivity in cancer treatment is increasing [58,59]. In this study, we found that ELSR and ERSR do not affect the viability of healthy cells using PBMC as a model, cells that could be affected during treatment with chemotherapeutic agents during the treatment of leukemias. Since IC_50_ was not obtained for this cell type, it is possible to infer that ELSR and ERSR exhibit at least 2-3-fold minimal selectivity for leukemic cells. This effect is desirable because the chemotherapeutic agents currently in use are not selective in their actions, causing side effects in the body, such as high toxicity in healthy cells [60]. Thus, we suggest that *S. rugosa* extracts possess potential therapeutic and chemopreventive effects.

Our results demonstrated that the extracts are more effective on the Jurkat cell line, a model of T-cell acute lymphoblastic leukemia. The differences found in our assays among the cell lines utilized might be attributable to mutations in each of the lines investigated. In acute lymphoblastic leukemias, such as Jurkat, the silencing of tumor suppressor and pro-apoptotic genes such as p53, PTEN, and BAX is common [15,61]. BCR-ABL1-positive leukemias, as in the K562 cell model, exhibit increased tyrosine kinase activity [13].

## 4. Materials and Methods

### 4.1. Chemicals and Reagents

The reagents 2,2-diphenyl-1-picrylhydrazyl (DPPH), 2,2′-azinobis 3-ethylbenzthiazoline-6-sulfonic acid (ABTS), 2,2′-Azobis(2-methylpropionamidine) dihydrochloride (AAPH), ascorbic acid (AA), bovine serum albumin (BSA), hydroxytoluene butylate (BHT), 3-(4,5-dimethylthiazol-2-yl)-2,5-diphenyltetrazolium bromide (MTT), ammonium persulfate, and potassium persulfate were purchased from Sigma-Aldrich (São Paulo, Brazil). Electrophoresis reagents were purchased from Bio-Rad Laboratories (São Paulo, Brazil). RPMI-1640 culture medium, antibiotics penicillin, streptomycin, neomycin, and fetal bovine serum (FBS) were purchased from Gibco/Invitrogen (Minneapolis, MN, USA). The other reagents used in this work were of analytical grade.

### 4.2. Plant Material

The leaves and roots of *S. rugosa* were collected in the city of Dourados, MS, Brazil (22°05′45″ S 55°20′46″ W), and permission to access genetic heritage (CTA) was registered in the Heritage SisGen under access code AA6FADF. An exsiccate specimen of the species was deposited in the Herbarium of the Universidade Federal da Grande Dourados with the registration number 4664. The leaves and roots of *S. rugosa* were washed with running water, dried in an oven with air circulation at 36 °C for 10 days, and ground in a knife mill. The leaf (ELSR) and root extracts of *S. rugosa* (ERSR) were prepared from 200 g of leaf or root powder macerated in 95% ethanol at a 1:7 (*w*/*v*) ratio at room temperature for 21 days.

Every seven days, the extract was filtered, the solvent was reserved, and a new aliquot of 95% ethanol was added to perform an exhaustive extraction of the compounds. The filtrates were pooled and concentrated in a rotary vacuum evaporator (Gehaka, São Paulo, SP, Brazil) at 37 °C. The filtrates were frozen and lyophilized to obtain the powders denoted as ELSR and ERSR. The yield of ELSR was 16.61%, and that of ERSR was 1%.

#### Phytochemical Analyses

The ELSR and ERSR were analyzed in a liquid chromatograph (UFLC, Shimadz, Kyoto, Japan) coupled to a diode array detector (DAD, Shimadzu) and to an electrospray ionization time-of-flight mass spectrometer (ESI-QTOF-micrOTOF QII, Bruker Daltonics, Billerica, MA, USA); positive ionization mode, with detection between 120–1200 Da. The sample was injected into a column C-18 (Kinetex, 2.6 μm, Phenomenex, Torrance, CA, USA). The chromatographic and mass spectrometric parameters applied were the same as those used by Castro et al. [19]. We identified the metabolites in the ELSR and ERSR by analyzing their molecular mass, fragmentation patterns, and ultraviolet (UV) absorption spectra, comparing these characteristics with the existing literature data. Whenever possible, we confirmed the identities of these compounds through comparative analysis with authentic standards.

### 4.3. Antioxidant Activity Assays

#### 4.3.1. Direct ABTS^•+^ Radical Scavenging Assay

The antioxidant capacity of ELSR and ERSR was assessed using an ABTS^•+^ assay [62]. A volume of 5 mL of ABTS solution (7 mM) and 88 μL of potassium persulfate solution (140 mM) were incubated for 12–16 h at room temperature in the dark. The ABTS^•+^ working solution was obtained by diluting the stock solution in distilled water, and its absorbance was determined (0.70 ± 0.05 units) at 734 nm in a spectrophotometer. ELSR or ERSR were prepared at different concentrations (0.1–100 μg/mL), and 20 μL was mixed with 1980 μL of the ABTS^•+^ working solution. The mixture was incubated for 6 min and absorbance was measured at 734 nm. Ascorbic acid (AA) and butylated hydroxytoluene (BHT) were used as reference antioxidants. A volume of 20 μL of distilled water was used for the determination of the absorbance of the negative control. The percentage of ABTS**^•+^** radical scavenging was calculated using the following Equation (1):

(1) $$\mathrm{Inhibition}\;\mathrm{of}\;\mathrm{ABTS}\left(\%\right)=\frac{\mathrm{Abs}\;\mathrm{control}-\mathrm{Abs}\;\mathrm{sample}}{\mathrm{Abs}\;\mathrm{control}}\;\times \;100$$

Three independent experiments were performed in triplicate and the concentration of the extracts needed for the capture of 50% of the ABTS**^•+^** radical (IC_50_) was determined, as well as the reference antioxidants.

#### 4.3.2. Direct DPPH^•^ Radical Scavenging Assay

The direct capture of the DPPH**^•^** radical by ELSR and ERSR was evaluated according to Gupta and Gupta [63]. For this, 200 μL of the ELSRs and ERSRs at different concentrations (0.1–100 μg/mL) were diluted in 1800 μL of 0.11 mM DPPH**^•^** solution prepared in 80% ethanol. The mixture was incubated for 30 min at room temperature in the dark. The absorbance at 517 nm was determined. Ascorbic acid and BHT were used as reference antioxidants. A 0.11 mM DPPH**^•^** solution plus 200 μL of 80% ethanol was used as the negative control. Three independent experiments were performed in triplicate and the percentage of direct DPPH**^•^** radical scavenging was calculated with Equation (2) as follows:

(2) $$Inhibition\;of\;DPPH\left(\%\right)=\frac{1-\left|sample\right|}{\left|negative\right|control}\times 100$$

The concentration of the extracts required for the capture of 50% of the DPPH**^•^** radical (IC_50_) was determined, as well as for the reference antioxidants.

The antioxidant activity assays were performed by diluting the extracts in a 10 mg/mL stock solution with 80% ethanol. From the stock solution, working solutions containing between 5 and 500 μg/mL were prepared in the solvents of each assay.

### 4.4. Protection against Oxidative Damage to the Macromolecules by ELSRs and ERSRs

#### 4.4.1. Azo Initiator 2,2′-Azobis-(2-amidinopropane) dihydrochloride AAPH-Induced Oxidation of Proteins

The protective effect of the extracts against AAPH-induced protein oxidation was evaluated according to the methodology of Mayo et al. [64], with minor modifications. A 3 μL aliquot of BSA (3 mg/mL, prepared in PBS) was pre-incubated with 3 μL of the ELSRs or ERSRs at different concentrations (5–500 μg/mL) for 30 min at 37 °C. Then, 3 μL of AAPH (120 mM, prepared in water) was added to the respective treatments, followed by further incubation for 120 min at 37 °C.

Then, 11 μL of sample buffer was added to each tube. The samples were incubated for 5 min at 95 °C and applied to a 12% SDS-PAGE polyacrylamide gel, separated at 200 V on the Mini-PROTEAN Tetra Cell system (Bio-Rad Laboratories). At the end of electrophoresis, the gels were stained, destained, and scanned in a Gel Doc EZ Imager (Bio-Rad Laboratories). The volume of the BSA bands was determined with the help of Image Lab software. An increased volume of the BSA bands was considered oxidative damage. BSA incubated with only AAPH was used as a positive control. The protection conferred by the different concentrations of the extracts was calculated by averaging four gels.

#### 4.4.2. Assay on Protection against DNA Damages (H_2_O_2_)

The protective effect of ELSR and ERSR against DNA fragmentation was evaluated using the method described by Kumar and Chattopadhyay [65] with modifications. A 4 μL aliquot of plasmid DNA (50 ng/μL) was incubated with 4 μL of ELSR or ERSR at different concentrations, diluted in PBS (5–500 μg/mL). Then, 4 μL of the oxidizing agent H_2_O_2_ at 30% was added to the respective treatments. The plasmid in the absence of UV light (CT), the plasmid in the presence of UV light (CTUV), the plasmid in the presence of the extracts plus UV light (ELSR or ERSR), and the control plasmid incubated only with hydrogen peroxide (H_2_O_2_) was used as the negative control. The effect of other reference antioxidants (rutin and catechin—150 μg/mL) on protecting DNA fragmentation was analyzed. The samples were incubated in a UVT-312 transilluminator at 302 nm at room temperature for 5 min. Then, the samples were applied to a 2% agarose gel containing ethidium bromide (10 mg/mL) and subjected to electrophoresis. Images of the gels were scanned using the Gel Doc™ EZ Imager photodocumentator (Bio-Rad Laboratories, Hercules, CA, USA) and analyzed using Image Lab™ software (version 6.0.1). The results of 3 gels were expressed as a percentage of DNA fragmentation.

The oxidative damage assays were performed by diluting the extracts in a stock solution containing 0.01% of 80% ethanol supplemented with PBS. From the stock solution, working solutions containing between 5 and 500 μg/mL were prepared in the solvents of each assay.

### 4.5. Cell-Based Assays

#### 4.5.1. Cell Culture

The human leukemia cell lines Jurkat (Human Acute Lymphoblastic Leukemia) and K562 (Human Chronic Myelogenous Leukemia) were kind donations by Professor Dr. Edgar J. Paredes-Gamero of the Department of Biochemistry, Universidade Federal de Sao Paulo, São Paulo, Brazil. The K562 and Jurkat were grown in suspension in RPMI 1640 media supplemented with 10 nM 4-(2-hydroxyethyl) piperazine-1-ethanesulfonic acid (HEPES), 24 nM sodium bicarbonate, 10% fetal bovine serum (FBS), and antibiotic Penicillin-streptomycin (10,000 U/mL), all from Gibco/Invitrogen, Minneapolis, MN, USA. All cells were kept in an incubator with a humidified atmosphere containing 5% CO_2_ at 37 °C.

#### 4.5.2. Isolation of Mononuclear Cells from Human Peripheral Blood

The Research Ethics Committee of the Centro Universitário da Grande Dourados (UNIGRAN) approved blood collection from a healthy non-smoking donor, process number 123/12. Human peripheral blood mononuclear cells (PBMC) were obtained by centrifugation using Ficoll-Paque-1077 Premium reagent (1.073 g/cm^3^). PBMC were suspended in RPMI-1640 medium supplemented with 20% SFB and 1% antibiotic and maintained at 37 °C in a humidified atmosphere containing 5% CO_2_.

#### 4.5.3. Evaluation of Cytotoxicity of ELSR and ERSR

The potential cytotoxicity of ELSR and ERSR was evaluated with the MTT reagent against leukemic cells (K562 and Jurkat) and PBMC. The K562, Jurkat (2 × 10^4^ cells/well), and PBMC (12 × 10^4^ cells/well) cells were plated in 96-well microplates and treated with different concentrations of the ELSR and ERSR (0–500 μg/mL solubilized in RPMI-1640 medium) for 24 and 48 h. After treatment, the microplates were centrifuged at 1200 rpm for 5 min and the culture medium was removed. A volume of 100 μL of MTT (0.5 mg/mL, prepared in RPMI-1640 medium) was added to the wells, and a 4 h incubation at 37 °C was performed. The plates were centrifuged, the medium was removed, and 100 μL of dimethyl sulfoxide (DMSO) was added to solubilize the formazan crystals. A control treatment was performed with 0.1% ethanol, the volume of solvent used for preparing the extracts. The absorbances were determined at 630 nm in a microplate reader (TP READER NM, Thermo Plate, Beijing, China) and cell viability was calculated using the following Equation (3):

(3) $$\mathrm{Cell}\;\mathrm{viability}\;\left(\%\right)=\frac{\left|treated\right|cells}{\left|control\right|-treatedcells}\times 100$$

The cell viability assay was performed by diluting the extracts in a stock solution containing 0.1% ethanol supplemented with culture medium.

### 4.6. Statistical Analyses

Data were presented as mean ± standard error of the mean (SEM). Statistically significant differences between groups were analyzed using the Student *t*-test for comparison between two groups and analysis of variance (ANOVA), followed by Dunnett’s test, for comparison of two or more groups, using Prism 7 Software (GraphPad Software, San Diego, CA, USA). The results were considered significant at *p* < 0.05. To establish the half-maximal inhibitory concentration (IC_50_) of DPPH and ABTS free radical scavenging, the samples were tested in serial dilutions (0.1–100 μg/mL) and to test the cytotoxicity against leukemic strains with different concentrations of the ELSR and ERSR (0–500 μg/mL), both were analyzed using nonlinear regression using GraphPad Prism 7 software. (GraphPad Software, San Diego, CA, USA). The results were considered significant at *p* < 0.05.

## 5. Conclusions

In this study, we demonstrated that ELSR and ERSR present flavonoid and naphthopyranone derivatives related to the observed antioxidant activities, capable of promoting direct radical scavenging and the protection of macromolecules against oxidative damage. In addition, the ELSR and ERSR exhibited cytotoxicity to human leukemia cell lines without affecting the viability of healthy cells. Our results expand the chemical compounds identified in the leaves and roots of *S. rugosa* and their pharmacological potential in the management of conditions related to oxidative stress and leukemia.

## Figures and Tables

**Table 1:** Chemical profile of ethanolic extracts of the leaves (ELSR) and roots (ERSR) of *S. rugosa* analyzed using UFLC-MS.

ELSR							
Compound	Classes	MS/MS	[M + H]^+^	Formula	UV	Time (min)	Peak
Catechin	Flavan-3-ol	-	291.087	C_15_H_14_O_6_	280	10.6	L-1
Rutin	Flavonol	465 (C_21_H_21_O_12_), 449 (C_21_H_21_O_11_), 303 (C_15_H_11_O_7_)	611.1599	C_27_H_30_O_16_	256/353	19.4	L-2
3,3′,5,5′-Tetrahydroxy-4-methoxystilbene	Stilbene	225 (C_14_H_9_O_3_), 213 (C_13_H_9_O_3_), 197 (C_13_H_9_O_2_), 185 (C_12_H_9_O_2_), 157 (C_11_H_9_O)	275.0914	C_15_H_14_O_5_	313	19.8	L-3
Epiafzelechin-epiafzelechin derivative	Proanthocyanidin	393 (C_22_H_17_O_7_), 285 (C_16_H_13_O_5_), 271 (C_15_H_11_O_5_), 259 (C_14_H_11_O_5_), 241 (C_14_H_9_O_4_), 147 (C_9_H_7_O_2_)	547.158	C_30_H_26_O_10_	280	20.1	L-4
Epiafzelechin-epiafzelechin derivative	Proanthocyanidin	393 (C_22_H_17_O_7_), 271 (C_15_H_11_O_5_), 241 (C_14_H_9_O_4_)	547.1582	C_30_H_26_O_10_	280	20.3	L-5
Epiafzelechin-epiafzelechin derivative	Proanthocyanidin	409 (C_22_H_17_O_8_), 269 (C_16_H_13_O_4_), 243 (C_14_H_11_O_4_), 163 (C_9_H_7_O_3_)	547.1589	C_30_H_26_O_10_	280	20.7	L-6
Kaempferol 3-*O*-rutinoside	Flavonol	449 (C_21_H_21_O_11_), 287 (C_15_H_11_O_6_)	595.1651	C_27_H_30_O_15_	265/347	21.1	L-7
Trihydroxyflavan-epiafzelechin derivative	Proanthocyanidin	393 (C_22_H_17_O_7_), 269 (C_16_H_13_O_4_)	531.1644	C_30_H_26_O_9_	280	22.6	L-8
Trihydroxyflavan-epiafzelechin derivative	Proanthocyanidin	393 (C_22_H_17_O_7_), 269 (C_16_H_13_O_4_)	531.1648	C_30_H_26_O_9_	280	24.9	L-9
Trihydroxyflavan-epiafzelechin derivative	Proanthocyanidin	393 (C_22_H_17_O_7_), 269 (C_16_H_13_O_4_), 243 (C_14_H_11_O_4_), 207 (C_11_H_11_O_4_)	531.1648	C_30_H_26_O_9_	280	25.6	L-10
Luteolin	Flavone	241 (C_14_H_9_O_4_), 153 (C_7_H_5_O_4_)	287.0546	C_15_H_10_O_6_	277/345	25.9	L-11
Trihydroxyflavan-epiafzelechin derivative	Proanthocyanidin	-	531.1651	C_30_H_26_O_9_	280	26.3	L-12
3-metoxyluteolin		-	317.0662	C_16_H_12_O_7_	279/350	27.2	L-13
Trimeric procyanidins	Proanthocyanidin	-	787.236	C_45_H_38_O_13_	280	29.2–30.1	L-14
Trimeric procyanidins	Proanthocyanidin	-	771.2412	C_45_H_38_O_12_	280	30.2–30.9	L-15
Dimeric tetrahydroanthracene derivative	Tetrahydroanthracene	533 (C_32_H_37_O_7_), 461 (C_29_H_33_O_5_)	593.275	C_34_H_40_O_9_	406	43.2	L-16
**ERSR**							
**Compound**	**Classes**	**MS/MS**	**[M + H]^+^**	**Formula**	**UV**	**Time (min)**	**Peak**
unknown	-	489 (C_24_H_25_O_11_), 423 (C_20_H_23_O_10_), 345 (C_18_H_17_O_7_), 291 (C_15_H_15_O_6_)	561.1791	C_24_H_32_O_15_	253/298	9.8	R-1
Obtusichromoneside derivative	Chromone	323 (C_16_H_19_O_7_), 293 (C_15_H_17_O_6_), 235 (C_12_H_11_O_5_), 205 (C_11_H_9_O_4_)	443.1542	C_20_H_26_O_11_	254/299	12.7	R-2
Obtusichromoneside derivative	Chromone	371 (C_20_H_19_O_7_), 259 (C_14_H_11_O_5_), 235 (C_12_H_11_O_5_), 205 (C_11_H_9_O_4_)	443.1548	C_20_H_26_O_11_	252/295	13	R-3
2-Methoxystypandrone	Naphthoquinone	197 (C_13_H_9_O_2_), 169 (C_12_H_9_O)	261.0757	C_14_H_12_O_5_	320	14.9	R-4
Tetrahydroxy-methoxy stilbene derivative	Stilbene	213 (C_13_H_9_O_3_), 197 (C_13_H_9_O_2_), 169 (C_12_H_9_O)	275.091	C_15_H_14_O_5_	313	16.2	R-5
3,3′,5,5′-Tetrahydroxy-4-methoxystilbene	Stilbene	225 (C_14_H_9_O_3_), 213 (C_13_H_9_O_3_), 197 (C_13_H_9_O_2_), 185 (C_12_H_9_O_2_), 157 (C_11_H_9_O)	275.0914	C_15_H_14_O_5_	313	19.8	R-6
chromone deriative	Chromone	271 (C_15_H_11_O_5_), 243 (C_14_H_11_O_4_), 203 (C_11_H_7_O_4_)	303.0867	C_16_H_14_O_6_	280/334	22	R-7
Putative norrubrofusarin gentiobioside	Naphthopyrone	259 (C_14_H_11_O_5_)	553.1554	C_25_H_28_O_14_	278/326/400	22.3	R-8
Putative hexahydroxy Flavanonol pentosyl-hexosyl	Flavanone	273 (C_15_H_13_O_5_)	567.1699	C_26_H_30_O_14_	279/312/366	22.9	R-9
Rubrofusarin gentiobioside	Naphthopyrone	273 (C_15_H_13_O_5_)	597.18	C_27_H_32_O_15_	277/324/399	24.2	R-10
Putative hexahydroxy Flavanonol sulfate	Flavanone	273 (C_15_H_13_O_5_), 230 (C_13_H_10_O_4_)	353.0314	C_15_H_12_O_8_S	276/310/369	25.1	R-11
Cassiaside B	Naphthopyrone	273 (C_15_H_13_O_5_)	567.1709	C_26_H_30_O_14_	277/324/395	26.4	R-12
Putative hexahydroxy-methoxy Flavanonol pentosyl-hexosyl	Flavanone	449 (C_22_H_25_O_10_), 287 (C_16_H_15_O_5_)	581.1864	C_27_H_32_O_14_	285/320/379	26.7	R-13
Rubrofusarin-O-glucopyranoside	Naphthopyrone	273 (C_15_H_13_O_5_)	435.1286	C_21_H_22_O_10_	277/324/401	27.5	R-14
unknown	-	449 (C_22_H_25_O_10_), 287 (C_16_H_15_O_5_)	611.1963	C_28_H_34_O_15_	279/330/413	28.6	R-15
Putative hexahydroxy-methoxy Flavanonol sulfate	Flavanone	287 (C_16_H_15_O_5_)	367.0472	C_16_H_14_O_8_S	285/321/377	28.9	R-16
unknown	-	449 (C_22_H_25_O_10_), 419 (C_21_H_23_O_9_), 287 (C_16_H_15_O_5_)	581.1861	C_27_H_32_O_14_	258/278/331/411	30.1	R-17
unknown	-	287 (C_16_H_15_O_5_)	449.1433	C_22_H_24_O_10_	279/330/411	30.8	R-18
Rubrofusarin	Naphthopyrone	230 (C_13_H_10_O_4_)	273.0758	C_15_H_12_O_5_	277/325/402	35.7	R-19
unknown	-	272 (C_15_H_12_O_5_), 254 (C_15_H_10_O_4_), 244 (C_14_H_12_O_4_), 226 (C_15_H_10_O_3_), 198 (C_13_H_10_O_2_)	287.0917	C_16_H_15_O_5_	281/337/425	37.7	R-20
unknown	-	561 (C_34_H_41_O_7_)	621.308	C_36_H_44_O_9_	410	43.4	R-21
unknown	-	561 (C_34_H_41_O_7_)	621.3077	C_36_H_44_O_9_	410	44.5	R-22

**Table 2:** IC_50_ values and maximum activity of the ELSR, ERSR, AA, and BHT.

Samples	ABTS^•+^			DPPH^•^		
	IC_50_ (µg/mL)	Maximum Activity (%)	(µg/mL)	IC_50_ (µg/mL)	Maximum Activity (%)	(µg/mL)
AA	1.32 ± 0.04	99.28 ± 0.16	5	2.35 ± 0.34	94.88 ± 0.16	10
BHT	6.08 ± 0.77	98.98 ± 0.24	50	71.86 ± 2.32	93.16 ± 0.23	500
ELSR	4.86 ± 0.51	98.15 ± 0.53	50	19.98 ± 1.96	95.24 ± 0.20	100
ERSR	8.33 ± 0.90	98.40 ± 0.33	25	13.37 ± 1.05	94.98 ± 0.27	100

Values are expressed as mean ± SEM (*n* = 3). AA = ascorbic acid; BHT = butylated hydroxytoluene.

**Table 3:** IC_50_ values were found for human leukemia cell lines K562 and Jurkat after treatment with ELSR and ERSR.

Cell Line	ELSR IC_50_ (μg/mL)		ERSR IC_50_ (μg/mL)	
	24 h	48 h	24 h	48 h
PBMC	ND	ND	ND	ND
K562	345.01 ± 2.53	242.54 ± 2.38	257.49 ± 2.41	223.00 ± 2.34
Jurkat	255.33 ± 2.40	171.45 ± 2.25	256.65 ± 2.40	189.30 ± 2.27

Values are expressed as mean ± SEM (*n* = 3). ND = IC_50_ could not be determined.

## Data Availability

The experimental data used to support the results of this study are included in the article.
